# 2,4-Disulfanyl-6-[(*E*)-(2-sulfanylbenz­yl)imino­meth­yl]phenol

**DOI:** 10.1107/S160053680903205X

**Published:** 2009-08-19

**Authors:** Yong-Ming Cui, Xi-Bin Dai, Lei Lei, Qing-Fu Zeng

**Affiliations:** a Engineering Research Center for Clean Production of Textile Dyeing and Printing, Ministry of Education, Wuhan 430073, People’s Republic of China

## Abstract

In the title compound, C_14_H_13_NOS_3_, the dihedral angle between the benzene rings is 73.26 (5)° and an intra­molecular O—H⋯N hydrogen bond occurs.

## Related literature

For background, see: Shi *et al.* (2007 ▶). For reference structural data, see: Allen *et al.* (1987 ▶);
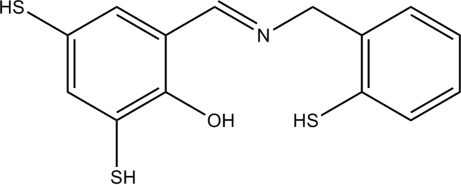


## Experimental

### 

#### Crystal data

C_14_H_13_NOS_3_
*M* *_r_* = 307.43
Monoclinic, 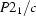

*a* = 11.9763 (13) Å
*b* = 8.2333 (13) Å
*c* = 14.2213 (13) Å
β = 98.723 (3)°
*V* = 1386.1 (3) Å^3^
*Z* = 4
Mo *K*α radiation
μ = 0.52 mm^−1^
*T* = 296 K
0.28 × 0.25 × 0.25 mm

#### Data collection

Enraf–Nonius CAD-4 diffractometer
Absorption correction: ψ scan (North *et al.*, 1968 ▶) *T*_min_ = 0.867, *T*_max_ = 0.880
7137 measured reflections
2443 independent reflections
1929 reflections with *I* > 2σ(*I*)
*R*_int_ = 0.025

#### Refinement

*R*[*F* ^2^ > 2σ(*F* ^2^)] = 0.047
*wR*(*F* ^2^) = 0.140
*S* = 1.06
2443 reflections
176 parameters
H-atom parameters constrained
Δρ_max_ = 0.28 e Å^−3^
Δρ_min_ = −0.39 e Å^−3^

### 

Data collection: *CAD-4 Software* (Enraf–Nonius, 1989 ▶); cell refinement: *CAD-4 Software*; data reduction: *XCAD4* (Harms & Wocadlo, 1995 ▶); program(s) used to solve structure: *SHELXS97* (Sheldrick, 2008 ▶); program(s) used to refine structure: *SHELXL97* (Sheldrick, 2008 ▶); molecular graphics: *SHELXTL* (Sheldrick, 2008 ▶); software used to prepare material for publication: *SHELXTL*.

## Supplementary Material

Crystal structure: contains datablocks global, I. DOI: 10.1107/S160053680903205X/hb5045sup1.cif

Structure factors: contains datablocks I. DOI: 10.1107/S160053680903205X/hb5045Isup2.hkl

Additional supplementary materials:  crystallographic information; 3D view; checkCIF report

## Figures and Tables

**Table 1:** Hydrogen-bond geometry (Å, °)

*D*—H⋯*A*	*D*—H	H⋯*A*	*D*⋯*A*	*D*—H⋯*A*
O1—H1⋯N1	0.82	1.87	2.591 (3)	147

**Fig. 1.**
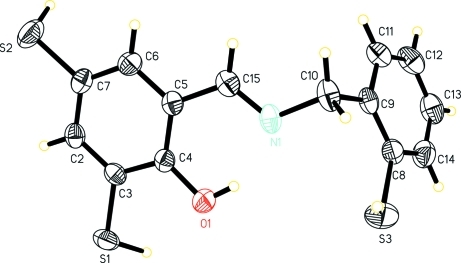
The molecular structure of (I) showing 30% probability displacement ellipsoids.

## supplementary crystallographic information

### Comment

There has been much research interest in Schiff base compounds due to their biological activities (Shi *et al.*, 2007). In this work, we report here the crystal structure of the title compound, (I). In (I), all bond lengths are within normal ranges (Allen *et al.*, 1987) (Fig. 1). There ais an intramolecular O—H···N hydrogen bond (Table 1) in (I). The dihedral angle between the two benzene rings is 73.26 (0.05) °.

### Experimental

A mixture of 2-hydroxy-3,5-disulfanylbenzaldehyde (186 mg, 1 mmol) and 2-(aminomethyl)benzenethiol (139 mg, 1 mmol) in methanol (10 ml) was stirred for 2 h. After keeping the filtrate in air for 6 d, yellow blocks of (I) were formed.

### Refinement

All H atoms were positioned geometrically (C—H = 0.93–0.97 Å, S—H = 1.20Å) and refined as riding with *U*_iso_(H) = 1.2*U*_eq_(carrier) or 1.5*U*_eq_(methyl C).

### Crystal data

**Table:**

C_14_H_13_NOS_3_	*F*(000) = 640
*M**_r_* = 307.43	*D*_x_ = 1.473 Mg m^−^^3^
Monoclinic, *P*2_1_/*c*	Mo *K*α radiation, λ = 0.71073 Å
Hall symbol: -P 2ybc	Cell parameters from 25 reflections
*a* = 11.9763 (13) Å	θ = 9–12°
*b* = 8.2333 (13) Å	µ = 0.52 mm^−^^1^
*c* = 14.2213 (13) Å	*T* = 296 K
β = 98.723 (3)°	Block, yellow
*V* = 1386.1 (3) Å^3^	0.28 × 0.25 × 0.25 mm
*Z* = 4	

### Data collection

**Table:**

Enraf–Nonius CAD-4 diffractometer	2443 independent reflections
Radiation source: fine-focus sealed tube	1929 reflections with *I* > 2σ(*I*)
graphite	*R*_int_ = 0.025
ω/2θ scans	θ_max_ = 25.0°, θ_min_ = 1.7°
Absorption correction: ψ scan (North *et al.*, 1968)	*h* = −14→10
*T*_min_ = 0.867, *T*_max_ = 0.880	*k* = −9→9
7137 measured reflections	*l* = −16→16

### Refinement

**Table:**

Refinement on *F*^2^	Primary atom site location: structure-invariant direct methods
Least-squares matrix: full	Secondary atom site location: difference Fourier map
*R*[*F*^2^ > 2σ(*F*^2^)] = 0.047	Hydrogen site location: inferred from neighbouring sites
*wR*(*F*^2^) = 0.140	H-atom parameters constrained
*S* = 1.06	*w* = 1/[σ^2^(*F*_o_^2^) + (0.0746*P*)^2^ + 0.5508*P*] where *P* = (*F*_o_^2^ + 2*F*_c_^2^)/3
2443 reflections	(Δ/σ)_max_ = 0.002
176 parameters	Δρ_max_ = 0.28 e Å^−^^3^
0 restraints	Δρ_min_ = −0.39 e Å^−^^3^

### Special details

**Table:**

Geometry. All e.s.d.'s (except the e.s.d. in the dihedral angle between two l.s. planes) are estimated using the full covariance matrix. The cell e.s.d.'s are taken into account individually in the estimation of e.s.d.'s in distances, angles and torsion angles; correlations between e.s.d.'s in cell parameters are only used when they are defined by crystal symmetry. An approximate (isotropic) treatment of cell e.s.d.'s is used for estimating e.s.d.'s involving l.s. planes.
Refinement. Refinement of *F*^2^ against ALL reflections. The weighted *R*-factor *wR* and goodness of fit *S* are based on *F*^2^, conventional *R*-factors *R* are based on *F*, with *F* set to zero for negative *F*^2^. The threshold expression of *F*^2^ > σ(*F*^2^) is used only for calculating *R*-factors(gt) *etc*. and is not relevant to the choice of reflections for refinement. *R*-factors based on *F*^2^ are statistically about twice as large as those based on *F*, and *R*- factors based on ALL data will be even larger.

### Fractional atomic coordinates and isotropic or equivalent isotropic displacement parameters (Å^2^)

**Table:**

	*x*	*y*	*z*	*U*_iso_*/*U*_eq_	
C2	0.3001 (2)	0.0386 (3)	−0.0207 (2)	0.0502 (6)	
H2	0.2415	−0.0310	−0.0118	0.060*	
C3	0.3723 (2)	0.0988 (3)	0.05602 (18)	0.0468 (6)	
C4	0.46151 (19)	0.2023 (3)	0.04542 (18)	0.0441 (6)	
C5	0.4777 (2)	0.2435 (3)	−0.04744 (18)	0.0448 (6)	
C6	0.4044 (2)	0.1839 (3)	−0.1250 (2)	0.0532 (7)	
H6	0.4148	0.2118	−0.1864	0.064*	
C7	0.3166 (2)	0.0837 (3)	−0.1110 (2)	0.0538 (7)	
C8	0.9011 (2)	0.3750 (3)	0.1048 (2)	0.0551 (7)	
C9	0.8496 (2)	0.3907 (3)	0.0113 (2)	0.0478 (6)	
C10	0.7427 (2)	0.4868 (3)	−0.0169 (3)	0.0605 (8)	
H10A	0.7476	0.5890	0.0174	0.073*	
H10B	0.7333	0.5106	−0.0844	0.073*	
C11	0.8989 (2)	0.3091 (3)	−0.0574 (2)	0.0594 (7)	
H11	0.8660	0.3151	−0.1209	0.071*	
C12	0.9966 (3)	0.2189 (4)	−0.0324 (3)	0.0718 (9)	
H12	1.0297	0.1668	−0.0792	0.086*	
C13	1.0442 (3)	0.2064 (4)	0.0608 (3)	0.0726 (9)	
H13	1.1092	0.1445	0.0772	0.087*	
C14	0.9971 (3)	0.2840 (4)	0.1300 (2)	0.0690 (8)	
H14	1.0297	0.2753	0.1934	0.083*	
C15	0.5736 (2)	0.3436 (3)	−0.0638 (2)	0.0523 (7)	
H15	0.5821	0.3710	−0.1257	0.063*	
N1	0.64557 (17)	0.3939 (3)	0.00480 (18)	0.0541 (6)	
O1	0.52860 (16)	0.2588 (2)	0.12252 (13)	0.0597 (5)	
H1	0.5820	0.3078	0.1060	0.090*	
S1	0.35256 (7)	0.04078 (11)	0.16895 (5)	0.0677 (3)	
H1A	0.4408	−0.0037	0.2128	0.102*	
S2	0.22437 (8)	0.01029 (13)	−0.20692 (7)	0.0860 (4)	
H2A	0.2734	−0.0823	−0.2515	0.129*	
S3	0.84366 (10)	0.47293 (14)	0.19417 (7)	0.0939 (4)	
H3A	0.8005	0.5977	0.1640	0.141*	

### Atomic displacement parameters (Å^2^)

**Table:**

	*U*^11^	*U*^22^	*U*^33^	*U*^12^	*U*^13^	*U*^23^
C2	0.0407 (14)	0.0441 (14)	0.0657 (18)	0.0040 (11)	0.0080 (12)	−0.0050 (12)
C3	0.0450 (14)	0.0445 (14)	0.0537 (15)	0.0098 (11)	0.0161 (11)	−0.0037 (11)
C4	0.0379 (13)	0.0410 (13)	0.0531 (15)	0.0074 (10)	0.0060 (11)	−0.0070 (11)
C5	0.0394 (13)	0.0392 (13)	0.0555 (15)	0.0099 (10)	0.0059 (11)	0.0044 (11)
C6	0.0543 (16)	0.0523 (15)	0.0514 (15)	0.0073 (12)	0.0029 (12)	0.0093 (12)
C7	0.0475 (15)	0.0527 (15)	0.0575 (17)	0.0041 (12)	−0.0037 (12)	−0.0010 (13)
C8	0.0483 (15)	0.0544 (16)	0.0643 (18)	−0.0054 (13)	0.0144 (13)	−0.0064 (13)
C9	0.0399 (13)	0.0369 (13)	0.0683 (17)	−0.0032 (10)	0.0136 (12)	0.0039 (11)
C10	0.0440 (15)	0.0444 (15)	0.095 (2)	0.0030 (11)	0.0151 (14)	0.0117 (14)
C11	0.0616 (17)	0.0542 (16)	0.0662 (18)	0.0002 (13)	0.0223 (14)	0.0053 (13)
C12	0.072 (2)	0.0588 (18)	0.094 (2)	0.0109 (15)	0.0409 (19)	−0.0026 (17)
C13	0.0478 (17)	0.067 (2)	0.105 (3)	0.0124 (15)	0.0171 (17)	0.0114 (19)
C14	0.0527 (17)	0.076 (2)	0.075 (2)	−0.0016 (15)	−0.0010 (15)	0.0072 (17)
C15	0.0477 (15)	0.0453 (14)	0.0651 (17)	0.0099 (12)	0.0121 (13)	0.0103 (12)
N1	0.0395 (12)	0.0460 (12)	0.0771 (16)	0.0029 (9)	0.0100 (11)	0.0029 (11)
O1	0.0542 (11)	0.0678 (13)	0.0566 (11)	−0.0068 (9)	0.0063 (9)	−0.0133 (9)
S1	0.0733 (5)	0.0826 (6)	0.0530 (5)	−0.0086 (4)	0.0280 (4)	−0.0057 (4)
S2	0.0784 (6)	0.1025 (7)	0.0678 (6)	−0.0198 (5)	−0.0192 (4)	−0.0078 (5)
S3	0.1002 (8)	0.1088 (8)	0.0779 (6)	0.0057 (6)	0.0300 (5)	−0.0335 (5)

### Geometric parameters (Å, °)

**Table:**

C2—C3	1.377 (4)	C10—N1	1.464 (3)
C2—C7	1.380 (4)	C10—H10A	0.9700
C2—H2	0.9300	C10—H10B	0.9700
C3—C4	1.392 (4)	C11—C12	1.386 (4)
C3—S1	1.726 (3)	C11—H11	0.9300
C4—O1	1.341 (3)	C12—C13	1.364 (5)
C4—C5	1.405 (4)	C12—H12	0.9300
C5—C6	1.391 (4)	C13—C14	1.365 (5)
C5—C15	1.461 (4)	C13—H13	0.9300
C6—C7	1.374 (4)	C14—H14	0.9300
C6—H6	0.9300	C15—N1	1.269 (3)
C7—S2	1.729 (3)	C15—H15	0.9300
C8—C14	1.374 (4)	O1—H1	0.8200
C8—C9	1.384 (4)	S1—H1A	1.2000
C8—S3	1.733 (3)	S2—H2A	1.2000
C9—C11	1.390 (4)	S3—H3A	1.2000
C9—C10	1.507 (4)		
			
C3—C2—C7	118.7 (2)	N1—C10—H10A	109.7
C3—C2—H2	120.7	C9—C10—H10A	109.7
C7—C2—H2	120.7	N1—C10—H10B	109.7
C2—C3—C4	122.3 (2)	C9—C10—H10B	109.7
C2—C3—S1	118.6 (2)	H10A—C10—H10B	108.2
C4—C3—S1	119.1 (2)	C12—C11—C9	120.7 (3)
O1—C4—C3	119.9 (2)	C12—C11—H11	119.7
O1—C4—C5	122.3 (2)	C9—C11—H11	119.7
C3—C4—C5	117.9 (2)	C13—C12—C11	120.1 (3)
C6—C5—C4	119.9 (2)	C13—C12—H12	119.9
C6—C5—C15	119.4 (2)	C11—C12—H12	119.9
C4—C5—C15	120.7 (2)	C12—C13—C14	120.5 (3)
C7—C6—C5	120.2 (3)	C12—C13—H13	119.7
C7—C6—H6	119.9	C14—C13—H13	119.7
C5—C6—H6	119.9	C13—C14—C8	119.3 (3)
C6—C7—C2	121.1 (2)	C13—C14—H14	120.4
C6—C7—S2	120.5 (2)	C8—C14—H14	120.4
C2—C7—S2	118.4 (2)	N1—C15—C5	121.4 (3)
C14—C8—C9	122.3 (3)	N1—C15—H15	119.3
C14—C8—S3	118.2 (2)	C5—C15—H15	119.3
C9—C8—S3	119.5 (2)	C15—N1—C10	118.5 (3)
C8—C9—C11	117.1 (2)	C4—O1—H1	109.5
C8—C9—C10	122.8 (3)	C3—S1—H1A	109.5
C11—C9—C10	120.0 (3)	C7—S2—H2A	109.5
N1—C10—C9	109.9 (2)	C8—S3—H3A	109.5

### Hydrogen-bond geometry (Å, °)

**Table:**

*D*—H···*A*	*D*—H	H···*A*	*D*···*A*	*D*—H···*A*
O1—H1···N1	0.82	1.87	2.591 (3)	147
